# Preparation of Gradient Polyurethane and Its Performance for Flexible Sensors

**DOI:** 10.3390/polym16121617

**Published:** 2024-06-07

**Authors:** Chuanqi Ning, Depeng Gong, Lili Wu, Wanyu Chen, Chaocan Zhang

**Affiliations:** School of Materials Science and Engineering, Wuhan University of Technology, Wuhan 430070, China; 317515@whut.edu.cn (C.N.); gdp@whut.edu.com (D.G.); polym_wl@whut.edu.cn (L.W.); chenwanyu@whut.adu.cn (W.C.)

**Keywords:** polyurethane, gradient, flake silver powder, composite, sensors

## Abstract

Flexible sensors are prone to the problems of slow recovery rate and large residual strain in practical use. In this paper, a polyurethane functional composite with a gradient change in elastic modulus is proposed as a flexible sensor to meet the recovery rate and residual strain without affecting the motion. Different hard and soft segment ratios are used to synthesize a gradient polyurethane structure. The conductive percolation threshold was obtained between 45 wt% and 50 wt% of flake silver powder. Both gradient polyurethane and gradient polyurethane composites demonstrated that gradient materials can increase the recovery rate and reduce residual strain. The gradient polyurethane composites had a tensile strength of 3.26 MPa, an elastic modulus of 2.58 MPa, an elongation at break of 245%, a sensitivity coefficient of 1.20 at 0–25% deformation, a sensitivity coefficient of 11.38 at 25–75% deformation, a rate of recovery of 1.95 s at a time, and a resistance to fatigue (over 1000 cycles at a fixed strain of 20% showed a stable electrical response). The sensing performance under different cyclic strain frequencies was also investigated. The process has practical applications in the field of wearable skin motion and health monitoring.

## 1. Introduction

With the development of electronic information and Internet technology, flexible wearable electronic products have attracted widespread attention [1,2,3,4,5]. Flexible sensors are currently used in motion-related fields such as electronic products [6,7], human interaction [8,9], and electronic skin [10,11]. Flexible strain sensors are generally composed of flexible substrates and conductive fillers [12,13], where the conductive fillers generally include two-dimensional graphene [14], carbon nanotubes [15], MXene materials [16], and metal nanowires [17,18]; and the flexible substrates generally include polyimide (PI) [19,20], hydrogel [21,22], polyure-thane (PU) [23,24], polydimethylsiloxane (PDMS) [25] and polystyrene-poly(ethylene-butylene)-polystyrene block copolymer (SEBS) [26].

Xia et al. [27] investigated the preparation of conductive hydrogels by micelle copolymerization under continuous stirring at room temperature. The tensile strength of the conductive hydrogel was 1.37 MPa, elongation at break was as high as 2058%, the modulus of elasticity was 0.053 MPa, and the residual strain under 90% cyclic strain reaches 60%. The recovery rate was 12 s at a time (50% strain), and the sensitivity coefficient was 5.44. Wang et al. [28] developed a foam-shaped strain sensor consisting of Ti_3_C_2_T_x_MXene, multi-walled carbon nanotubes (MWCNTs) and thermoplastic polyure-thane (TPU), which has a modulus of elasticity of 0.14 MPa, elongation at break of 140%, and the recovery rate was 5 s at a time (80% strain), capable of cycling 2000 times. Fang et al. [29] used a polyurethane substrate with a polyaniline conductive polymer (PU-DA-1/1-PANI) to form an interpenetrating synergistic dual-network conductive hydrogel with an elongation at break of 500%, and a modulus of elasticity of 0.3 MPa. The recovery rate was 3.34 s at a time (25% strain) and the substrate is capable of cycling 1200 times with a sensitivity factor of 2.89. Amjadi et al. [30] fabricated a flexible sensor based on nanocomposites of silver nanowires (AgNW) and PDMS elastomers with an elastic mod-ulus of 3.5 MPa, elongation at break of 70%, and a residual strain of 2% at 10% cyclic strain. The recovery rate was 3 s at a time (10% strain), and the sensor had a sensitivity factor of 5. Zhou et al. [31] fabricated strain sensors by coating SEBS fibers sequentially with dopamine (PDA) and carboxylated multi-walled carbon nanotubes (MWCNTs), with a tensile strength of 2.39 MPa, a modulus of elasticity of 11.67 MPa, elongation at break was 626.92%, the residual strain was nearly 10% at 50% cyclic tensile strain, and the recovery rate was 2 s at a time (40% strain), capable of cycling 2500 times. Yang et al. [32] prepared graphene/polyimide foam compression sensors using dip-coating polyimide (PI) foam templates, followed by chemical reduction and thermal reduction, and the compressive strains of prepared foams were less than 5%. The modulus of elasticity was 0.005 MPa, when at 17% cyclic strain its residual strain was 8%, the recovery rate was 5 s at a time (17% strain), and it was capable of compression cycling 1000 times.

The systems with high sensitivity reported in the above literature have a very large modulus of elasticity, which is obviously problematic as a motion sensor. The system with small modulus of elasticity has a long recovery time, which is not suitable for the detection of the general movement frequency of the human body. As a flexible sensor, the recovery rate should be large to match the motion frequency of the monitored object. In addition, the tensile strength and modulus of elasticity should be small, and the deformation stress should not affect the motion. Therefore, it is an important research direction to study a flexible sensor that simultaneously takes into account the fast recovery rate and small elastic modulus.

This study is based on the current problem of a single material with a high modulus of elasticity that has a fast recovery rate and may affect motion, and a single material with a low modulus of elasticity that has a slow recovery rate and may not be able to monitor motion effectively. A step-gradient polyurethane functional composite material with gradient change in elastic modulus is designed as a flexible sensor, aiming to take into account the characteristics of fast recovery rate and small elastic modulus, so that the flexible sensor meets the requirements of sensitivity and deformation stress at the same time. The mechanical and sensing properties of the flexible sensor are also investigated.

## 2. Materials and Methods

### 2.1. Chemicals and Reagents

**Figure 1:**
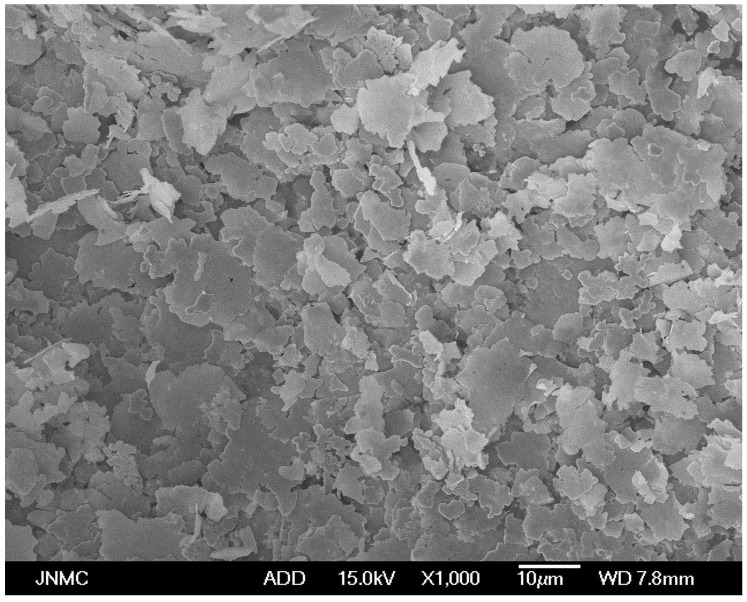
SEM image of the flake silver powder.

Polytetrahydrofuran (PTMG = 2000) and polycaprolactone (PCL = 2000) were purchased from Xuzhou Tactile Yang New Material Co., Ltd., Xuzhou, China. Isophorone diisocyanate (IPDI) and trimethylolpropane (TMP) were purchased from Shanghai McLean Biochemical Technology Co., Ltd., Shanghai, China. Methylthiobenzene diamine (DMTDA) was purchased from Shandong Orilon Chemical Co., Ltd., Qingdao, China. 4,4-Methylene-bis(3-chloro-2,6-diethyl) (MCDEA) was purchased from Aladdin Reagent Co., Ltd., Shanghai, China. Figure 1 shows the research grade micron flake silver powder (99.99%, 5~10 μm), purchased from XinDrill Alloy Material Co., Ltd., Shanghai, China. Acetone was purchased from Aladdin Reagent Co., Ltd., Shanghai, China.

### 2.2. Preparation of Gradient Polyurethane and Gradient Polyurethane Composites

Bottom polyurethane will be PTMG-2000 at 110 °C under vacuum dehydration for 2 h and 60 °C, to add isophorone diisocyanate (IPDI) and catalyst Dibutyltin dilaurate (DBTDL). This was followed by a reaction at 80 °C for two hours after the addition of chain expander Dimethylthiomethyltoluene diamine (DMTDA) and a crosslinking agent Trimethylolpropane (TMP) reaction for half an hour, while being hot poured into molds at room temperature (25 °C). This was followed by semi-curing for 12 h, Table 1 shows the proportion of the bottom layer of polyurethane formulations. The middle layer of polyurethane is made of PTMG-2000 and PCL-2000, the rest of the steps are the same, and it is finally poured onto the half-cured bottom layer of polyurethane and half-cured for 12 h. Table 2 shows the proportion of the middle layer of polyurethane formulations. The top polyurethane layer uses the chain extender 4,4-methylene-bis(3-chloro-2,6-diethyl) (MCDEA), the rest of the steps are the same, and it is finally poured on the half-cured middle polyurethane layer to cure for 24 h. Table 3 shows the proportion of the top polyurethane layer formulation to obtain the gradient polyurethane. The -NCO/-OH ratio of the whole reaction system is 1.05. The synthesis of stepped-gradient polyurethane is performed by preparing each layer of polyurethane and then curing it layer by layer. The thickness of the bottom, middle and top layers of polyurethane is the same as the thickness of the gradient polyurethane.

**Figure 2:**
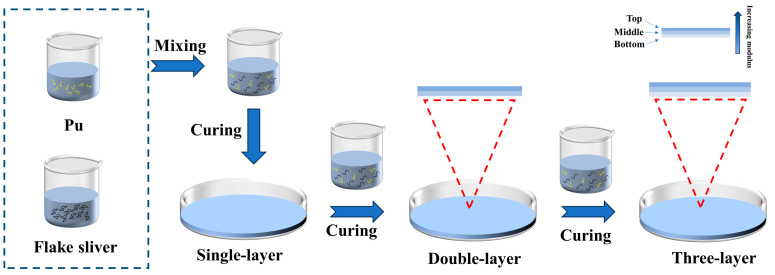
Preparation process of gradient polyurethane composites.

The experimental scheme of washing flake silver powder is shown in Table 4. After washing the flake silver powder with acetone solution of acetic acid, weighing different mass fractions of flake silver powder (35~70%, each increase of 5% as a specimen) in a beaker, adding polyurethane, stirring for 10 min to make it basically free of bubbles, then pouring it into molds, the curing method is the same as that of gradient polyurethane curing method. The schematic of the synthesis of gradient polyurethane composites is shown in Figure 2.

### 2.3. Tensile Testing (Stress–Strain Curves)

The tensile test was carried out using a tensile compression testing machine (WDW-L02, Meister, Qingdao, China). According to GB/T13022-91 standard, for each layer of polyurethane mechanical properties, cyclic tensile test is conducted, tensile speed is set to 500 mm/min, and the data of each specimen are measured and recorded.

### 2.4. Attenuated Total Reflection Fourier Transform Infrared Spectroscopy (ATR-FTIR)

The surface functional group structure of polyurethane elastomers was characterized by attenuated total reflection Fourier transform infrared spectroscopy (Nicolet, Madison, WI, USA) and the polyurethane elastomer structure was analyzed.

### 2.5. Recovery Time and Residual Strain Testing

**Figure 3:**
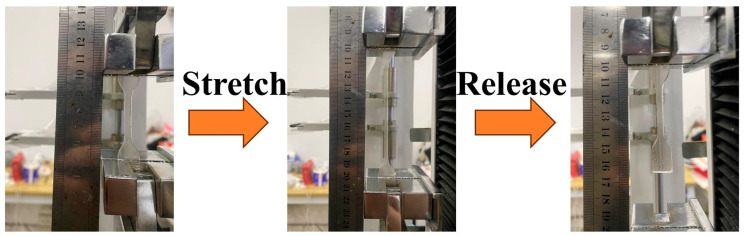
Schematic diagram of recovery time and residual strain testing.

The polyurethane layers and gradient polyurethane specimens were placed on a tensile compression testing machine (WDW-L02, Meister, Qingdao, China) for fixed stretching to 50% and then released, and the recovery time as well as the residual strain were recorded. The experimental operation of recovery time and residual strain is shown in Figure 3.

### 2.6. Sensing Performance

**Figure 4:**
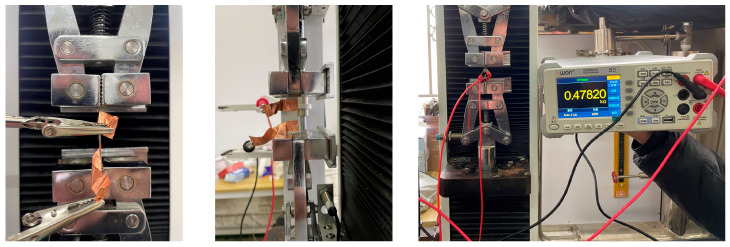
Diagram of strain sensing experimental setup.

Sensing performance was tested using a tensile compression tester (WDW-L02; Meister, Qingdao, China) and a benchtop multimeter (HT8808A, Lilliput Optoelectronics, Fuzhou, China). Strain sensing performance is assessed through the tensile compression testing machine each time the tensile strain increased by 5%. Then, the resistance value stabilizes, and the resistance value of the desktop multimeter is read, revealing a tensile compression rate of 50 mm/min. Different cyclic strain frequency is seen in the 50% fixed strain conditions, through the tensile testing machine being continuously set up at different cyclic tensile rates for the 5 cycles, and the resistance value of the desktop multimeter being read. The flowchart of the strain measurement setup is shown in Figure 4.

## 3. Results

As a flexible sensor, the requirements of both sensitivity and elastic modulus need to be balanced. Since a single material with a high modulus of elasticity has a fast recovery rate, it may affect the motion; a material with a low modulus of elasticity has a slow recovery rate and may not be able to monitor the motion effectively. In this manuscript, a polyurethane functional composite with a gradient change in modulus of elasticity is investigated.

### 3.1. Preparation of Gradient Polyurethanes and Polyurethane Layers with Properties of Tensile Strength and Modulus of Elasticity

**Figure 5:**
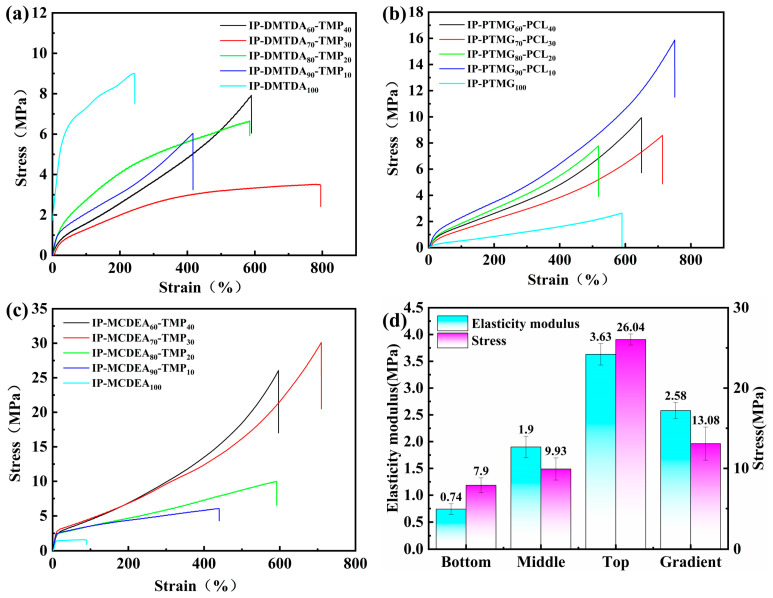
Stress–strain curves: (**a**) bottom layer; (**b**) middle layer; (**c**) top layer; (**d**) modulus of elasticity and tensile strength.

In order to study the sensors that simultaneously balance the small elastic modulus and fast recovery rate, a gradient polyurethane system with a total of three layers decreasing the elastic modulus layer by layer from the outside to the inside was designed. The more flexible polytetrahydrofuran polyol and dimethylsulfanyltoluene diamine were synthesized to form a low modulus bottom layer, a moderate modulus middle layer was synthesized with polycaprolactone polyol and dimethylsulfanyltoluene diamine, and a top layer with a high modulus of elasticity was synthesized with polytetrahydrofuran polyol and 4,4-methylene-bis(3-chloro-2,6-diethyl) with a bis(aryl) ring structure. The proportions of the layers were reconciled the proportional solutions were filtered to meet requirements. The experimental results are shown in Figure 5 and Table 5. The bottom layer polyurethane was synthesized using the chain extender DMTDA and the crosslinker TMP, and the ratios of DMTDA and TMP ranged from 100/0 to 60/40, and the results are shown in Figure 5a, when the ratio of DMTDA to TMP was 60/40, at this time, the tensile strength was 7.9 ± 0.92 MPa, the modulus of elasticity was 0.74 ± 0.12 MPa, and the elongation at break was 590 ± 46.32%. Polyester polyol PCL and polyether polyol PTMG were used to synthesize the middle layer polyurethane. The ratio of PTMG to PCL was adjusted, and in Figure 5b, when the ratio of PTMG to PCL was 90/10, the tensile strength was 9.93 ± 1.38 MPa, the modulus of elasticity was 1.90 ± 0.15 MPa, and the elongation at break was 629 ± 33.09%. The top polyurethane layer was synthesized using the chain extender MCDEA and the cross-linker TMP, and the ratio of MCDEA and TMP was adjusted, and in Figure 5c, when the ratio of MCDEA to TMP was 60/40, the tensile strength was 26.04 ± 0.69 MPa, the modulus of elasticity was 3.63 ± 1.01 MPa, and the elongation at break was 597 ± 26.88%. The gradient polyurethane was synthesized after selecting the ratio of each layer of polyurethane, and the tensile strength of gradient polyurethane was 13.08 ± 2.06 MPa, modulus of elasticity was 2.58 ± 0.85 MPa, and elongation at break was 416 ± 39.84%. Figure 5d shows that the gradient polyurethane was prepared by forming a progressive gradient change in elastic modulus with respect to tensile strength. The top layer has the highest modulus of elasticity because the addition of rigid aromatic rings to the top layer increases the proportion of hard segments. The middle layer has a higher modulus of elasticity than the bottom layer because polycaprolactone polyols are not as flexible as polytetrahydrofuran polyols, and the increase in rigidity increases the modulus of elasticity. The reduced elongation at break of the gradient polyurethane may be related to the strength of the three-layer interfacial bond. Although they are all polyurethane materials, the intermolecular forces between the interfaces affect the mechanical properties of the overall gradient material.

**Figure 6:**
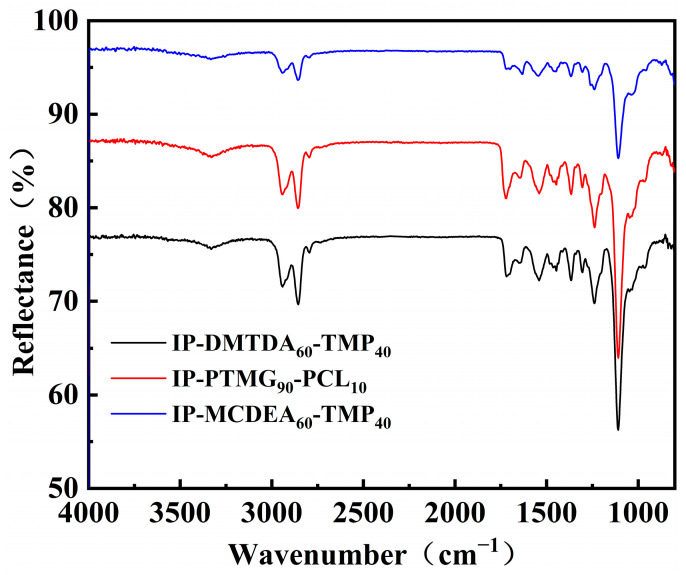
Infrared spectra of bottom, middle and top polyurethane layers.

After the preparation of each layer of gradient polyurethane, the structure of each layer was further characterized. The chemical structure of the polyurethane specimens was verified by attenuated total reflection Fourier transform infrared spectroscopy (ATR-FTIR) analysis. As shown in Figure 6, the peak near 830 cm^−1^ corresponds to the C-H out-of-plane bending vibration on the benzene ring, and the peaks near 3320 cm^−1^ and 1549 cm^−1^ correspond to the urethane groups and the -NH stretching and bending vibrations in DMTDA and MCDEA. The peaks of methyl and methylene stretching vibrations appeared at 2856 cm^−1^ and 2942 cm^−1^. The peak at 1110 cm^−1^ was the stretching vibration of C-O-C of polyether polyol in the soft segment. It indicates that DMTDA and MCDEA were effectively introduced into the polymer backbone of the polyurethane. The disappearance of the typical-NCO peaks near 2260~2280 cm^−1^ marks the completion of the reaction system. The preparation results showed agreement with the expected design results. The middle layer introduces more ester groups compared to the bottom layer, resulting in better mechanical properties than the bottom layer. The MCDEA introduced in the top layer is a chain extender with more aromatic rings than the bottom layer, and the aromatic ring structure will greatly improve the mechanical properties of the elastomer. The stepped gradient polyurethane materials with gradually increasing modulus of elasticity were further screened.

### 3.2. Recovery Time and Residual Strain of Gradient Polyurethane with Layers of Polyurethane

**Figure 7:**
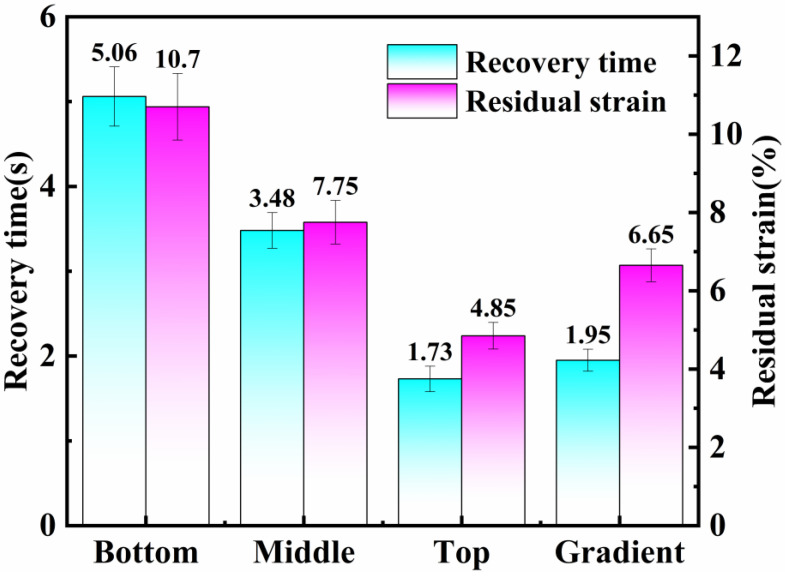
Recovery time and residual strain at 50% fixed tension for each layer and gradient polyurethane.

The recovery rate and residual strain of the gradient composite layer and each layer of polyurethane affect the motion sensing performance of the flexible sensors, and each layer of polyurethane and gradient polyurethane were fixed stretched to 50% and then released, and the recovery time as well as the residual strain were recorded. The experimental results are shown in Figure 7. The recovery rates of the bottom, middle, top and gradient polyurethanes were 5.06 s/time, 3.48 s/time, 1.73 s/time, 1.95 s/time, and the residual strains were 10.7%, 7.75%, 4.85%, and 6.65%, respectively. Recovery time and residual strain reflect the resilience of polyurethane elastomers. The resilience of polyurethane is related to factors such as crosslink density. The higher the crosslink density the more it restricts the movement of the molecular chains, making the polyurethane more resilient. The difference between the middle layer and the bottom layer is the introduction of more ester groups in the hard section, which forms more cross-linking points with the soft section, resulting in better resilience. The difference between the upper layer and the bottom layer is the introduction of MCDEA, a double benzene ring structural chain extender, into the hard section, which forms more cross-linking points with the soft section and improves the resilience. It can be seen that the larger the modulus of elasticity, the faster the recovery rate and the smaller the residual strain. The experimental results show that the recovery rate and residual strain of the gradient polyurethane is between the bottom and top layer, probably due to the resilience in the top layer leading to the overall reduction in recovery rate and residual strain.

### 3.3. Conductivity of the Bottom Polyurethane as a Function of the Mass Fraction of Silver Flake Powder

**Figure 8:**
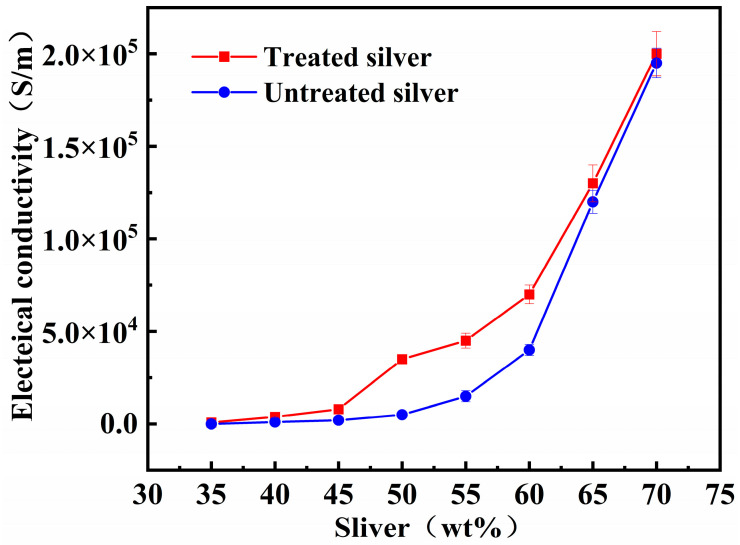
Relationship between conductivity and mass fraction of silver flake powder.

In order to obtain conductive polyurethane with good electrical properties and to determine the conductive percolation threshold of the system, the relationship between the conductivity of the underlying polyurethane composites and the mass fraction of the flake silver powder was investigated, and the effect of the untreated and acetone solution of acetic acid treatment of the flake silver powder on the conductivity was also studied. The experimental results are shown in Figure 8. The experimental results show that the conductivity increased after cleaning the flake silver powder with acetone solution of acetic acid, because the surface of the flake silver powder prepared by the manufacturer had a lot of stabilizers leading to a decrease in conductivity. As the content of flake silver powder increased, the conductivity gradually increased. When the mass fraction reaches 45%, the conductivity increases significantly, indicating that the conductive percolation threshold is obtained between 45% and 50%. Flake silver powders are interconnected to form conductive pathways. When the percolation threshold before the conductive filler is not completely intact, the formation of continuous conductive channel is less likely. The electron is mainly channeled through the field emission and tunneling effect to realize the transmission—at this time the conductivity is low. When the system reaches the conductive threshold, the conductive filler completes full contact, the electrons are mainly channeled through the conductive channel for transmission, and conductivity suddenly increases. Therefore, 45% mass fraction of flaky silver powder was chosen as the experimental scheme to further test and analyze the mechanical and sensing properties of polyurethane composites.

### 3.4. Mechanical Properties of and Ag/Gradient Polyurethane Composites

**Figure 9:**
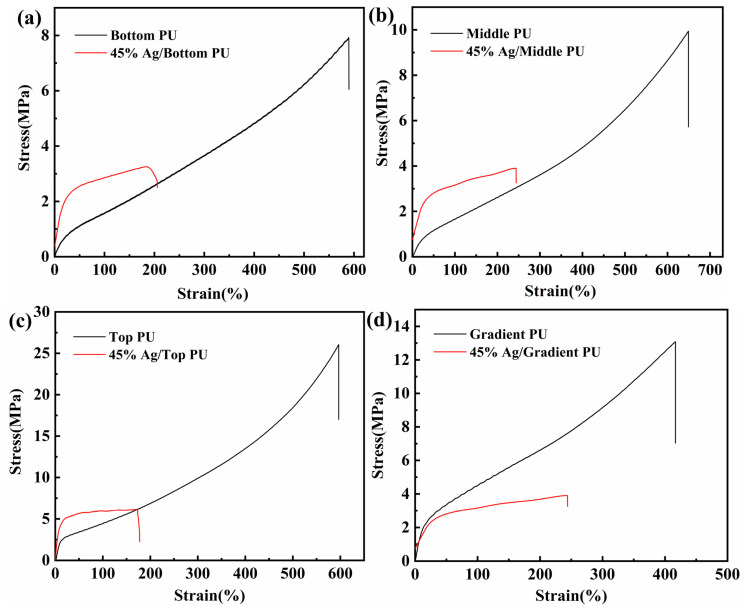
Stress–strain curves of composites: (**a**) bottom layer; (**b**) middle layer; (**c**) top layer; (**d**) gradient.

Strain tensile response properties are an important property of sensors, and the mechanical properties of polyurethane and polyurethane composites were investigated because the addition of conductive fillers to polymer systems may affect the mechanical properties. Flaky silver powder with a mass fraction of 45% was added to each layer of polyurethane. From Figure 9a, the tensile strength, modulus of elasticity and elongation at break of the bottom polyurethane were 7.94 ± 0.92 MPa, 0.74 ± 0.12 MPa and 590 ± 46.32%, and those of the polyurethane composite were 3.25 ± 1.56 MPa, 2.81 ± 0.55 MPa and 185 ± 48.54%. In Figure 9b, the middle polyurethane layer had a tensile strength, modulus of elasticity and elongation at break of 9.93 ± 1.38 MPa, 1.90 ± 0.15 MPa and 629 ± 33.09%, and the polyurethane composite had a tensile strength, modulus of elasticity and elongation at break of 3.91 ± 1.48 MPa, 4.12 ± 0.52 MPa and 243 ± 62.73%. In Figure 9c, the top polyurethane layer had its tensile strength, modulus of elasticity and elongation at break of 26.04 ± 0.69 MPa, 3.63 ± 1.01 MPa and 597 ± 26.88%, and the polyurethane composites had their tensile strength, modulus of elasticity and elongation at break of 6.08 ± 1.07 MPa, 6.42 ± 1.56 MPa and 173 ± 49.88%. In Figure 9d, the gradient polyurethane shows tensile strength, elastic modulus and elongation at break of 13.08 ± 2.06 MPa, 2.58 ± 0.85 MPa and 416 ± 39.84%, respectively, and the gradient polyurethane composite’s tensile strength, elastic modulus and elongation at break were 3.74 ± 1.35 MPa, 6.16 ± 1.02 MPa and 248 ± 55.46%. Although the tensile strength and elongation at break decreased significantly after the addition of flake silver powder, the modulus of elasticity increased significantly, which can accelerate the recovery rate and reduce the residual strain. The addition of conductive fillers restricts the movement of the molecular chain segments, resulting in an increase in the modulus of elasticity. The modulus of elasticity of the gradient polyurethane composite is higher than that of the bottom layer, and this result is consistent with the structure we designed. However, the modulus of elasticity of the gradient polyurethane composite is lower than that of the top layer because the thickness of the top layer is only one-third of the original gradient polyurethane composites for flexible sensor applications.

### 3.5. Strain Sensing Performance

**Figure 10:**
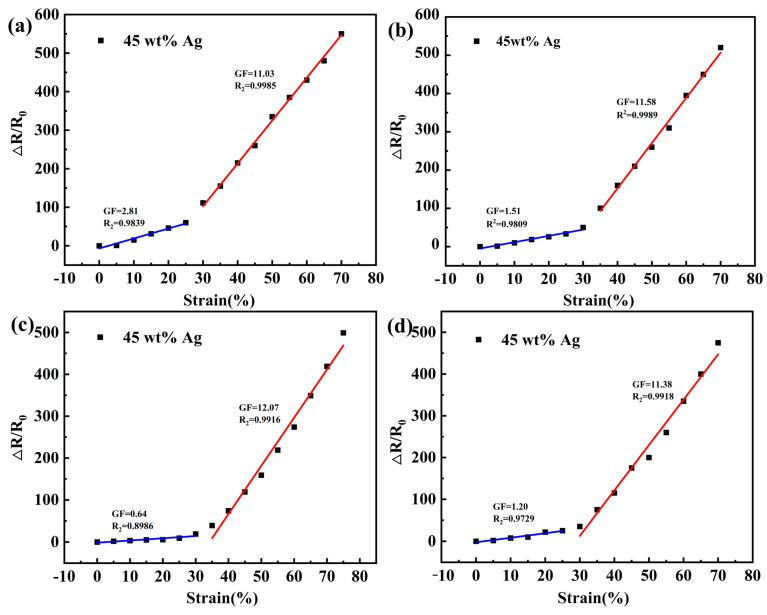
Fitted curves of strain versus relative resistance: (**a**) bottom layer; (**b**) middle layer; (**c**) top layer; (**d**) gradient composite layer.

In order to investigate the strain sensing performance of the sensors, a series of data points corresponding to the resistance values were tested for every 5% tensile strain, the variation of resistance with applied strain was investigated for the bottom, middle, top and gradient composites. GF is a metric representation of strain sensitivity defined as GF = (ΔR/R_0_)/ε, ΔR = R − R_0_, R and R_0_ are the test and initial resistance of the specimen, respectively, and ε is the strain [33,34,35]. The resistance of polyurethane composites increases with strain, and the change in relative resistance (ΔR/R_0_) with strain is shown in two stages: When the tensile deformation is small (strain less than 30%), the distance between the conductive particles is very small, the electrons are mainly transmitted through the conductive channel, a continuous conductive network is formed, and the relative resistance rate of change is small. When the tensile deformation exceeds 30%, the distance between the conductive particles changes a lot, the distance between the conductive particles changes a lot, the chance of forming a continuous conductive channel is small, the electrons are mainly transmitted through the field emission and tunneling effect, and the relative resistance rate of change is large. The experimental results of the bottom polyurethane composite are shown in Figure 10a. In the first stage of the bottom polyurethane composite (strain 0~25%), the ΔR/R_0_ increases gradually, and the slope of the curve is 2.81 for the GF value; in the second stage (strain > 20%), the GF reaches 11.03. The experimental results of the middle polyurethane composite are shown in Figure 10b. In the first stage of the middle polyurethane composite (strain 0~30%), the GF is 1.51, and in the second stage (30~70%), the GF is 11.58. The experimental results of the top polyurethane composite are shown in Figure 10c. The first stage (strain 0~30%) GF = 0.64 and the second stage (30~70%) GF = 12.07, while the gradient polyurethane composite has a first stage (strain 0~30%) GF = 1.2 and a second stage (30~70%) GF = 11.38, as shown in Figure 10d. The gradient polyurethane composite has a sensitivity between the bottom and the top because the flexible modulus of elasticity of the substrate varies in a gradient, and the high modulus of elasticity of the top polyurethane composite provides sensitivity and recoverability, which satisfies both the sensitivity and deformation stress requirements. The current hydrogel system reported in the literature has a sensitivity of 5.44, a recovery time of 12 s/time, and a modulus of elasticity of 0.053 MPa, which may affect the sensing response. In comparison, the dopamine (PDA) and carboxylated multi-titanium-walled carbon nanotubes (MWCNTs) coated SEBS fiber system has a sensitivity of 3717, a recovery time of 2 s/time, and a modulus of elasticity of 11.67 MPa. In contrast, the gradient urethane composites that we prepared have a sensitivity of 11.38, a recovery time of 1.95 s, and a modulus of elasticity of 6.16 MPa. Both sensitivity and deformation stress are satisfied by gradient polyurethane composites.

### 3.6. Different Cyclic Strain Frequencies

**Figure 11:**
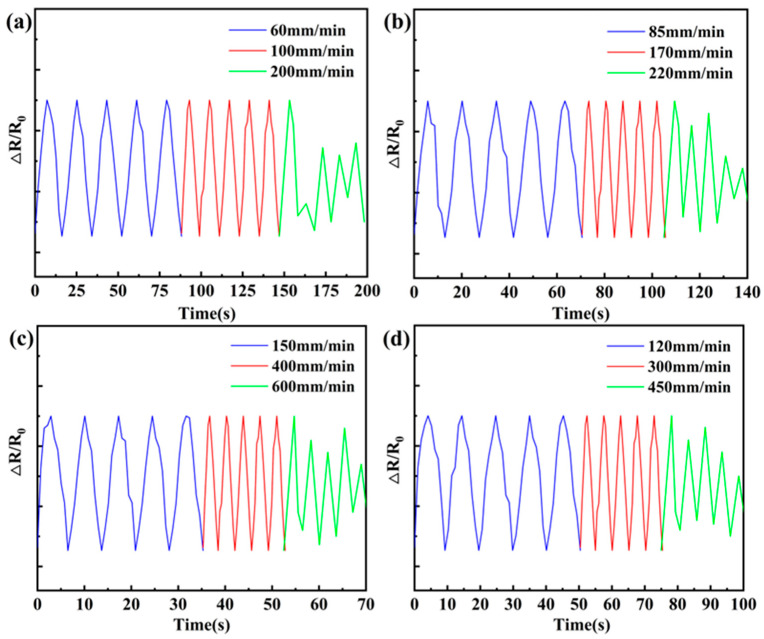
Cyclic strain frequency: (**a**) bottom; (**b**) middle; (**c**) top; (**d**) gradient.

The recovery rate of the flexible sensor matches the frequency of movement of the monitored object, but fewer studies have been reported. In order to study the recovery rate of polyurethane composites. Through the program control settings in a fixed 50% strain conditions, the tensile testing machine for continuous different cycles under the tensile frequency, each tensile frequency under the cycle of 5 times. In Figure 11a, the bottom layer has a uniform change in relative resistance (ΔR/R_0_) reacting to the recovery frequency coinciding with the stretching frequency when the stretching–compression frequency is set to 0.1 Hz and 0.15 Hz. When the tensile compression frequency is 0.2 Hz, the ΔR/R_0_ does not change uniformly in response to the recovery frequency not being consistent with the tensile frequency. In Figure 11b, in the middle layer, when the tensile compression frequency is set to 0.15 Hz and 0.3 Hz, the uniform change in ΔR/R_0_ responds to the recovery frequency being consistent with the tensile frequency. When the tensile compression frequency is 0.4 Hz, the ΔR/R_0_ does not change uniformly in response to the recovery frequency not being consistent with the tensile frequency. In Figure 11c, the top layer reacts to the uniform change in ΔR/R_0_ to recover the frequency in agreement with the tensile frequency when the tensile compression frequency is set to 0.3 Hz and 0.6 Hz. When the tensile compression frequency is 0.8 Hz, the ΔR/R_0_ unevenly varies in response to the recovery frequency being inconsistent with the tensile frequency. In Figure 11d, the gradient composite layer reacts to a uniform change in ΔR/R_0_ in response to a recovery frequency consistent with the tensile frequency when the tensile compression frequency is set to 0.2 Hz and 0.4 Hz. When the tensile compression frequency is set at 0.6 Hz, the uneven change in ΔR/R_0_ responds to the recovery frequency being inconsistent with the tensile frequency. When the stretching frequency is increased, its ΔR/R_0_ changes unevenly, which is because the recovery time of the polymer without the conductive filler does not satisfy its stretching frequency. It is shown that the recovery frequency of gradient polyurethane composites is between the top and bottom layers, and the recovery frequency is faster than that of the middle layer. The human body’s general walking motion frequency is about 0.5~2 Hz [36], and the gradient composites material prepared by us have a recovery frequency of about 0.5 Hz, which may be applied to the detection of human walking motion.

### 3.7. Fatigue Resistance

**Figure 12:**
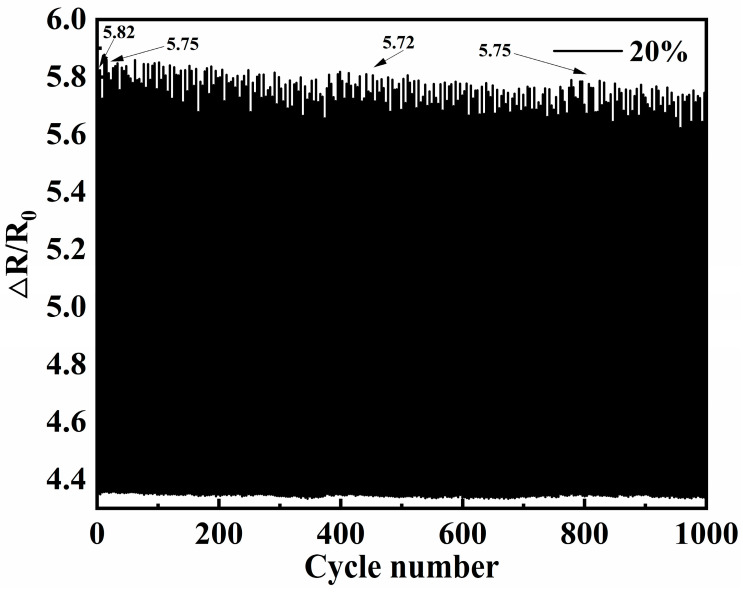
Cyclic durability curves of gradient polyurethane composites (20% strain).

Fatigue resistance is a very important indicator for strain sensors in practical applications. Flexible sensors based on gradient polyurethane materials exhibit excellent stability at loading and unloading rates of 50 mm/min (20% strain) for 1000 cycles. In Figure 12, the gradient polyurethane composites have ΔR/R_0_ of 5.82 at the 1st time, ΔR/R_0_ of 5.75 at the 20th time, and ΔR/R_0_ of 5.72 ± 0.03 at the 21th~1000th times. It can be seen that the curves basically do not have a significant attenuation after the 20th time, the attenuation before the 20th time may be due to the permanent deformation, and after the 21th time they are all in the range of 5.72 ± 0.03, which shows that good accuracy and repeatability. Regarding the performance for long-term use and higher number of cycles, it may be related to the aggregation of conductive fillers as well as the permanent deformation of the polymer, which will be further studied.

### 3.8. Human Motion Detection

**Figure 13:**
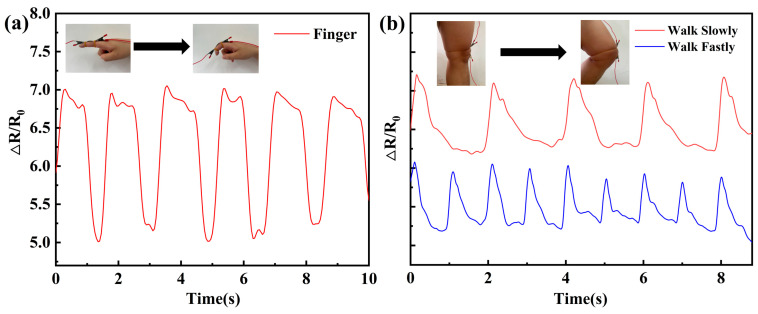
Human movement detection: (**a**) finger movement; (**b**) leg movement.

Gradient strain sensors are characterized by good flexibility, high sensitivity, and faster and more complete recovery, which can be matched with motion and applied to human wearable devices to monitor various human motions. The applications of gradient strain sensors are shown in Figure 13, and preliminary studies can apply the sensors to joint movements, which can be roughly categorized into small and large movements. As shown in Figure 13a, the gradient strain sensor detects small movements of the human body (finger joints with a strain of about 10%), and by placing the sensor on the finger joints at a uniform speed, an electrical signal can be detected at regular intervals. In Figure 13b, large motions of the human body (joint motions with a strain of 20% or more, such as running) are detected by placing the sensors on the knee joints for jogging and fast running, and the electrical signals are detected in real time on the knee joints for both jogging and fast running. The results of the electrical response to motion showed that the strain amplitudes were all within the range of sensing changes and had good responsiveness. The different peak shapes reflect the small differences in finger motion and need to be followed up with further modeling and analysis.

## 4. Conclusions

In this study, a functional polyurethane composite with gradient variation of elastic modulus was investigated to consider flexible sensors with fast recovery speed and small elastic modulus, and to fulfill the requirements of sensitivity and deformation stress. Gradient polyurethane composites have shown the ability to increase sensitivity, reduce residual strain and minimize deformation stress. The experimental results found that the tensile strength, elastic modulus and GF of the gradient polyurethane were closer to those of the middle layer, but the recovery time and residual strain were better than those of the middle layer. We believe that the recovery of the gradient structure as a motion sensor is still slow, and the next step will be to optimize the structure and thickness of each layer to achieve the optimization of the gradient structure. Also, anisotropic metal powders with anisotropic properties such as needles are used as conductive fillers, so as to further investigate the accuracy and recoverability of the sensors.

## Figures and Tables

**Table 1:** Formulation of bottom polyurethane (mmol).

Sample	IPDI	PTMG	DMTDA	TMP
IP-DMTDA_100_-TMP_0_	75	25	47.5	0
IP-DMTDA_90_-TMP_10_	75	25	42.75	3.17
IP-DMTDA_80_-TMP_20_	75	25	38	6.33
IP-DMTDA_70_-TMP_30_	75	25	33.25	9.5
IP-DMTDA_60_-TMP_40_	75	25	28.5	12.67

**Table 2:** Formulation of middle polyurethane (mmol).

Sample	IPDI	PTMG	PCL	DMTDA	TMP
IP-PTMG_100_-PCL_0_	75	25	0	28.5	12.67
IP-PTMG_90_-PCL_10_	75	22.5	2.5	28.5	12.67
IP-PTMG_80_-PCL_20_	75	20	5	28.5	12.67
IP-PTMG_70_-PCL_30_	75	17.5	7.5	28.5	12.67
IP-PTMG_60_-PCL_40_	75	15	10	28.5	12.67

**Table 3:** Formulation of top polyurethane (mmol).

Sample	IPDI	PTMG	DMTDA	TMP
IP-DMTDA_100_-TMP_0_	75	25	47.5	0
IP-DMTDA_90_-TMP_10_	75	25	42.75	3.17
IP-DMTDA_80_-TMP_20_	75	25	38	6.33
IP-DMTDA_70_-TMP_30_	75	25	33.25	9.5
IP-DMTDA_60_-TMP_40_	75	25	28.5	12.67

**Table 4:** Formulation of washing flake silver powder.

Mass of Polyurethane (g)	Mass of Flaky Silver Powder (g)	Acetone (mL)	Acetic Acid (mL)
2	0.6	15	3
2	0.7	15	3
2	0.8	15	3
2	0.9	15	3
2	1.0	15	3
2	1.1	15	3
2	1.2	15	3
2	1.3	15	3
2	1.4	15	3

**Table 5:** Mechanical properties of gradient composite layers and layers of polyurethane.

Sample	Tensile Strength (MPa)	Elongation at Break (%)	Modulus of Elasticity (MPa)
Bottom	7.9 ± 0.92 MPa	590 ± 46.32%	0.74 ± 0.12 MPa
Middle	9.93 ± 1.38 MPa	629 ± 33.09%	1.90 ± 0.15 MPa
Top	26.04 ± 0.69 MPa	597 ± 26.88%	3.63 ± 1.01 MPa
Gradient	13.08 ± 2.06 MPa	416 ± 39.84%	2.58 ± 0.85 MPa

## Data Availability

Data are contained within the article.
